# TESTING MULTICOMPONENT DIGITAL THERAPIES TO PROMOTE HEALTHIER LIFESTYLE BEHAVIORS IN OLDER ADULTS: A PILOT STUDY

**DOI:** 10.1093/geroni/igad104.1402

**Published:** 2023-12-21

**Authors:** Lorraine Evangelista, Andrew-Thomas Reyes, Dieu-My Tran, Reimund Serafica

**Affiliations:** University of California Irvine, Irvine, California, United States; University of Nevada, Las Vegas, Las Vegas, Nevada, United States; University of Nevada, Las Vegas, Las Vegas, Nevada, United States; UNLV, Las Vegas, Nevada, United States

## Abstract

Multicomponent digital interventions to encourage healthy diet and physical exercise in older persons at risk for cardiovascular disease are understudied. We compared the feasibility and effectiveness of Get FIT vs. Get FIT+, two digital-based therapies, to encourage healthier lifestyle behaviors in this high-risk population. Fifty-four older adults (66.6 ± 5.9 years, 61% females, 61% married, 50% Asians, 39% Whites) were randomized to Get FIT (n=24), which included a health app, an activity tracker, and one 45-minute behavioral counseling session on healthy eating and exercise, or Get Fit+ (n=30), which added three months of health coach-personalized text messaging. We assessed retention, engagement, satisfaction, diet, physical activity, weight, cardiometabolic risks, and psychological well-being pre-and post-intervention. At three months, both groups had a 100% retention rate. Get FIT+ participants expressed high satisfaction, with 92% reporting that they would recommend Get FIT+. Individuals in both groups increased their physical activity and reduced their caloric consumption from baseline to three months. However, these changes were significantly greater in the intervention group (p .0001). Get Fit+ participants averaged a three-pound weight loss, whereas their counterparts averaged a three-pound weight gain (p .0001). Get FIT+ individuals had significantly lower systolic and diastolic blood pressure, triglycerides, total cholesterol, and HgbAa1C than Get FIT participants from baseline to three months. Both groups showed similar psychological well-being over time. Our findings imply that the Get Fit+ intervention may be more effective in lowering cardiometabolic risk in this cohort, although longer-term trials are needed.

